# Qualitative study on the virtual reality-based empty-chair technique in middle-aged South Korean men

**DOI:** 10.3389/fpsyg.2025.1559171

**Published:** 2025-10-08

**Authors:** Kieun Yoo, Eunha Kim

**Affiliations:** Department of Psychology, Ajou University, Suweon, Republic of Korea

**Keywords:** gestalt therapy, empty-chair technique, virtual reality, middle-aged men, interpretative phenomenological analysis

## Abstract

**Introduction:**

The gestalt empty-chair technique facilitates dialog between clients and an imagined person or aspect of themselves to explore and resolve emotions or conflicts. Although it has proven to be therapeutic, middle-aged South Korean men may find its traditional format uncomfortable because of cultural norms and traditional gender roles that discourage emotional expression. This study explored the perceptions of a virtual reality (VR)-based adaptation of the empty-chair technique.

**Methods:**

Fourteen middle-aged South Korean men (aged 40–59 years) participated in sessions involving interaction with a virtual representation of their younger selves in a VR environment, followed by in-depth interviews. Data were analyzed using Smith’s Interpretative Phenomenological Analysis to reveal participants’ lived experiences.

**Results:**

Three positive experience domains emerged: “emotional reactions from meeting my childhood self,” “shifting attitudes toward the self,” and the “advantages of using VR.” Conversely, three negative experience domains were identified: “difficulty revisiting childhood without constraints,” “elements that disrupted immersion,” and “areas for improvement.”

**Discussion:**

The findings highlight the potential of the VR-based empty-chair technique as a counseling tool for middle-aged men, offering innovative ways to overcome the limitations of the traditional approach.

## 1 Introduction

Recent advancements in information technology (IT) and artificial intelligence (AI) have brought significant changes to psychological assessment and counseling (Hwang and Hwang, 2020). In this evolving landscape, virtual reality (VR) has emerged as an innovative tool for psychological intervention. VR enables users to experience computer-generated virtual environments as if they are real by stimulating their visual, auditory, and olfactory senses (McGrath et al., 2018; Seo and Lee, 2022). In VR-based counseling, clients typically wear head-mounted displays (HMD) to engage in virtual environments. Among its applications, VR exposure therapy (VRET) is the most widely used and has demonstrated effectiveness in treating various psychological disorders such as phobias, social anxiety, and post-traumatic stress disorder (Carl et al., 2019; Porras-Garcia et al., 2020; Van Bennekom et al., 2021; Wechsler et al., 2019). Compared with traditional exposure therapies, VRET offers vivid, controllable stimuli and a safe environment where therapists can tailor exposure intensity to client needs (Freitas et al., 2021; Kim and Kim, 2023; Rothbaum et al., 2001).

Beyond anxiety-related conditions, VR-based counseling has been applied to alcohol dependence, eating disorders, and social skills training (Simon et al., 2020; Riva et al., 2021; Langener et al., 2022). Recently, VR has also been adapted to experiential and humanistic approaches, including Gestalt therapy. Gestalt therapy emphasizes present-moment awareness and integration of fragmented experiences (Perls et al., 1951; Greenberg et al., 2008). A core intervention in Gestalt therapy is the empty-chair technique, which guides clients to conduct dialog with an imagined person (e.g., a family member or internal self-aspect) seated in an empty chair (Staemmler, 1995). Through this dialogic process, clients can explore unresolved emotions, gain insight, and enhance their self-acceptance (Greenberg et al., 2008; Paivio and Greenberg, 1995; Pugh and Salter, 2018).

However, the traditional empty-chair technique can often feel artificial, particularly in cultural settings where emotional disclosure is discouraged (Hall et al., 2011). VR-based adaptations help address these limitations by providing visible, embodied avatars in immersive environments, which make the dialog feel more concrete and less dependent on imagination (Brahnam, 2014; Ma et al., 2023). Prior studies have demonstrated the promise of this approach—for instance, bereaved clients expressing emotions toward a virtual spouse (Brahnam, 2014) and individuals engaging in dialog with their future selves (Ganschow et al., 2021). Together, these findings indicate that VR has the potential to enhance engagement and deepen emotional experience within Gestalt-based interventions.

Building on this line of work, the present study focuses specifically on dialog with a standardized younger-self avatar. Dialog with this avatar is intended to address unresolved emotional issues from earlier life stages—such as neglect, unmet needs, or internalized criticism—that often persist into adulthood as shame, self-criticism, or unresolved grief (Lee, 2013). In this study, the younger self was represented by a standardized child avatar, approximately 8–10 years old. Engaging in dialog with this virtual representation enabled participants to reflect on their past, cultivate self-compassion, and reframe earlier experiences. Rather than focusing only on symptom reduction, the intervention aimed to foster deeper outcomes, including reconciliation with one’s past and greater self-acceptance.

This focus is particularly relevant for middle-aged South Korean men, a group at heightened risk for depression, anxiety, and suicide (Health Insurance Review and Assessment Service, 2022; Statistics Korea, 2023). In this population, cultural norms that regard emotional expression as a sign of weakness often discourage these men from seeking help or sharing their struggles (Jung and Lee, 2021). Such barriers underscore the importance of therapeutic approaches that make emotional engagement feel safer and more acceptable. The immersive and embodied nature of VR provides such an approach. By presenting a visible virtual presence rather than relying on imagination alone, it helps externalize inner dialog and reduce self-consciousness. This may be especially beneficial in collectivist contexts where emotional restraint is valued and expressions of vulnerability are often stigmatized (Lee and Kim, 2024).

In summary, the present study explored how middle-aged South Korean men experience the VR-based empty-chair (VREC) technique. The VREC technique developed in this study uses a standardized virtual avatar representing a younger self, allowing participants to engage in inner dialog aimed at resolving unresolved emotional issues. In particular, the study focused on psychological distress rooted in childhood relationships with patriarchal and strict fathers, who have traditionally held the central authority role within South Korean families (Sung and Joeng, 2019). Such father–child relationships have been identified as a salient source of lasting emotional conflicts—including shame, self-criticism, and emotional inhibition—among men in adulthood (Jung and Lee, 2021; Rohner and Carrasco, 2014).

Accordingly, focusing on father–child relationships offers a culturally grounded rationale for investigating the potential of the VREC technique. Specifically, we examined the following three research questions: (1) What positive experiences did participants report? (2) What negative experiences did participants report? (3) How immersed were participants in the VREC experience? By addressing these questions, this study aimed to evaluate the therapeutic potential of VREC and its capacity to overcome the limitations of traditional empty-chair techniques in fostering emotional expression among middle-aged South Korean men.

## 2 Materials and methods

### 2.1 Research design

This qualitative study used a single-session design combining a VREC intervention with in-depth post-session interviews. The aim was to explore participants’ lived experiences of the intervention using Interpretative Phenomenological Analysis (IPA; Smith et al., 2009), which is well suited for examining how individuals make sense of emotionally significant experiences.

### 2.2 Participants

A purposeful sampling strategy was employed to recruit participants. Fourteen middle-aged men (aged 40–59 years) participated in this study. The average age of participants was 51.32 years (*SD* = 7.31). All participants reported experiencing psychological distress associated with their childhood relationships with their patriarchal and strict fathers. Psychological distress was assessed using a prescreening interview in which participants were asked to describe emotionally difficult memories involving their fathers. Individuals were included if they reported ongoing emotional difficulties related to these relationships, such as persistent resentment, sadness, helplessness, or regret. Commonly expressed feelings toward fathers include resentment, helplessness, and regret. While this focus may limit the generalizability of the findings, it reflects a culturally significant dynamic in South Korea, where rigid father-son relationships and traditional masculinity norms continue to shape men’s emotional development (Sung and Joeng, 2019). Exploring how these men engaged in emotion-focused techniques in a VR environment offers important insights into therapeutic approaches tailored to populations facing cultural barriers to emotional expression. Table 1 presents the detailed participant characteristics.

**Table 1:** Demographic characteristics of participants.

No.	Age	Occupation	Marital status	Highest education level
1	49	Baker	Married	High school graduate
2	40	High school teacher	Married	College graduate
3	47	Wallpaper installer	Married	College graduate
4	41	Office worker	Married	Master’s degree
5	58	Judicial scrivener	Married	Master’s degree
6	49	Engineer	Married	Master’s degree
7	55	Business owner	Married	College graduate
8	58	Security	Married	College graduate
9	55	Security	Married	College graduate
10	40	Unemployed	Married	College graduate
11	48	Engineer	Married	Doctoral degree
12	54	Counselor	Single	Master’s degree
13	52	Psychiatrist	Married	Doctoral degree
14	51	Lawyer	Divorced	Master’s degree

### 2.3 Study contexts

This study was approved by the Institutional Review Board of [University Name] (Approval #202402-HS-003). Participants were recruited through local online communities frequently used by middle-aged men in South Korea, including Carrot Market’s “Neighborhood Life” and Naver’s “Neighbor News.” Individuals with a known history of VR-related side effects (e.g., nausea or dizziness) were excluded. We selected the Carrot Market and Naver online communities for recruitment because they are commonly used by South Korean men in their 40s and 50s to anonymously and approachably discuss everyday life and personal concerns, making them suitable channels for reaching participants who may otherwise be reluctant to engage in face-to-face recruitment for emotional or psychological studies. All participants provided written informed consent, and confidentiality and anonymity were ensured.

### 2.4 Data collection tools

A VR system equipped with Meta Quest 2 HMDs was used to deliver the intervention. This standalone headset provided six degrees of freedom and was paired with handheld controllers to enable interactions. The virtual environment and virtual representation of a “virtual boy” were developed using Unity 2021.3.4f1, a cross-platform game engine widely used for immersive experiences. Participants engaged with a standardized younger-self avatar approximating the appearance of an 8–10-year-old child, represented both in physical appearance and contextual setting. The avatar’s behavior was preprogrammed to display subtle nonverbal cues such as a slumped posture, lowered head, avoidance of eye contact, and light fidgeting.

To assess participants’ experiences within the virtual environment, the I-group Presence Questionnaire (IPQ; Schubert, 2003) was administered. This instrument evaluates three key dimensions: presence (the subjective sense of “being there”), immersion (the level of engagement), and realism (the perceived authenticity of the environment). The IPQ consists of 11 items rated on a 7-point Likert scale (1 = very unlikely, 7 = very likely). In the present study, internal consistency was acceptable, with Cronbach’s *α* values of 0.81 for presence, 0.75 for immersion, and 0.80 for realism. Participants’ subjective experiences were further examined through in-depth interviews guided by a semi-structured protocol comprising seven open-ended questions (see Table 2). The protocol included prompts specifically designed to explore psychological distress associated with participants’ childhood experiences, particularly those involving their fathers. All interviews were conducted in Korean, audio recorded with participants’ consent, and transcribed verbatim for analysis.

**Table 2:** Interview protocol.

1. How much does the person sitting on the empty chair feel like your actual childhood self?
2. What was your experience meeting your childhood self in the virtual environment? How did you feel when encountering and speaking to your childhood self?
3. What factors hindered you from meeting and interacting with your childhood self in the virtual
4. Environment?
5. What changes or improvements do you think could make the experience better?
6. The traditional empty chair technique is conducted using imagery (imagination) techniques. Compared to the VR-based approach, which method do you think is better for immersion?
7. How do you think it would feel if the traditional approach had been used instead?
8. If you were to receive counseling using the empty-chair technique, which method would you prefer: the traditional approach or VR-based approach? Why?

### 2.5 Procedure

Each session lasted approximately 90 min and consisted of four sequential stages: rapport building and pre-intervention discussion, VREC intervention, questionnaire administration, and in-depth interviews (see Table 3). In the pre-intervention phase, participants engaged in a brief conversation with the researcher to build rapport and then guided to reflect on their childhood experiences, particularly their relationships with their fathers. Participants were asked to recall emotionally significant memories and consider messages they would like to communicate with their younger selves. Common themes included expressions of comfort, empathy, and reassurance, such as “You are not alone” or “You deserve love and respect.”

**Table 3:** Summary of the procedure.

Step	Description	Duration
1	Explanation of the study procedure and informed consent	5 min
2	Exploration of childhood experience	20 min
3	VR setup and instruction for devise use	5 min
4	VR empty-chair technique	20 min
5	In-depth interview	30 min
6	Ingroup Presence Questionnaire	5 min
7	Debriefing and Q&A	5 min
Total		90 min

**Figure 1:**
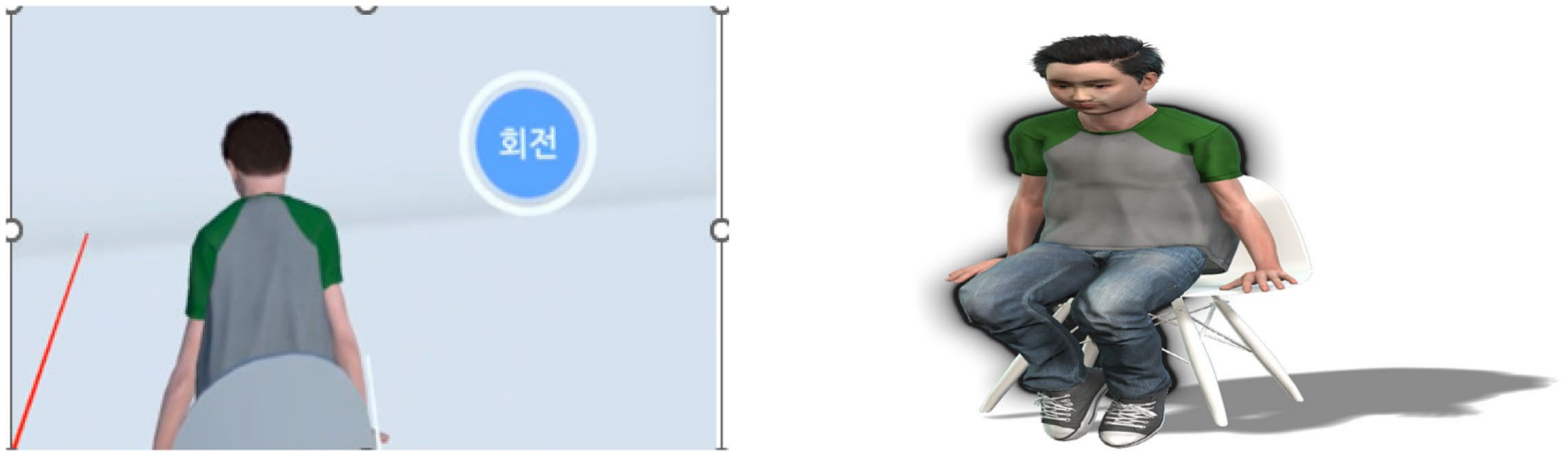
An example of the virtual body.

During the VR intervention, participants customized the virtual representation’s appearance, including clothing, hairstyle, and background setting (e.g., a room, kitchen, or classroom), to closely resemble their childhood environment. However, the core visual and behavioral features of the virtual representation were standardized across all sessions to ensure consistency. Specifically, the virtual representation was portrayed as a melancholic early teenager with a slouched posture, somber facial expressions, and minimal eye contact. These features were deliberately designed to evoke emotional themes common to participants’ childhood experiences with authoritarian fathers. The boy initially faced away and turned to face the participant when the “rotate” button was pressed, signaling readiness for emotional engagement. Once participants were ready to begin the dialog, they activated the virtual representation’s response by pressing a “rotate” button, at which point the figure turned to face them. Participants then engaged in self-directed communication with the avatar, expressing the thoughts and emotions they had previously identified. Although the specific content varied across individuals, common themes included reassurance, empathy, and acknowledgment of unmet needs. The researcher provided only minimal instructions (e.g., “You may begin now”) and refrained from intervening further, in order to preserve the authenticity of participants’ self-directed dialog. Figure 1 presents screenshots of the VR interface, including the standardized younger-self avatar (approximately 8–10 years old) and the rotate button that allowed participants to turn the avatar to face them and initiate dialog. Immediately after the VR session, participants completed the IPQ to assess their subjective sense of presence, immersion, and realism within the virtual environment.

Finally, semi-structured in-depth interviews were conducted in Korean to explore participants’ experiences with the VREC technique. All interviews were audio recorded, transcribed, and translated into English. The second author, fluent in both Korean and English, translated the transcripts, and an external bilingual auditor reviewed the translations to ensure accuracy and cultural fidelity. After the tenth interview, participants’ responses began to show significant overlap, and no new themes emerged following the twelfth interview, indicating thematic saturation. All participants’ data were anonymized using numerical identifiers to ensure confidentiality.

### 2.6 Data analysis

Descriptive statistics were used to analyze participants’ responses to the IPQ. Subsequently, in-depth interview data were examined using the three-step process of IPA outlined by Smith et al. (2009): engaging with the data, recording preliminary observations, identifying recurring themes, and making connections between those themes. The first and second authors independently reviewed each transcript multiple times to understand each participant’s experiences. Initial observations and reflections were documented at the transcript margins. Next, significant statements or “meaning units,” elements of a participant’s description that convey a specific aspect of their experience (Giorgi, 2009), were identified. These meaning units were then grouped into emerging themes based on the patterns and relationships in the data. While code frequencies were not formally counted, themes were identified based on recurring patterns of meaning across multiple participants, typically appearing in at least 3–4 transcripts. This approach ensured that each theme reflected shared experiential elements. Consistent with Smith et al. (2009), we prioritized the interpretative meaning of participants’ experiences over numerical frequency when organizing and refining themes.

To address potential researcher bias, both authors maintained individual reflexivity journals throughout the analysis. These journals document personal assumptions, emotional reactions, and interpretive decisions. During analytic meetings, we revisited these notes to bracket our own perspectives and ensure that theme development was grounded in participants’ lived experiences rather than shaped by our prior beliefs or theoretical expectations. In addition, the authors held repeated discussions to resolve interpretive differences, for instance, whether participants’ emotional responses reflected unresolved trauma or more generalized discomfort and reached consensus on the final themes through collaborative reflection.

An external auditor with extensive qualitative research experience, including IPA in counseling psychology, provided feedback on the emergent themes and subthemes. Based on these comments, the first and second authors reviewed the transcripts and revised the themes and subthemes. For example, we made minor edits to theme names and removed several themes that were only noted by one participant (e.g., “felt a sudden pride seeing how far I have come since childhood” or “had trouble initiating a conservation with a virtual boy”). These revisions ensured that the themes reflected shared experiences among participants. Finally, the participants were invited to review the themes and subthemes. Of the 14 participants, three responded and confirmed that the themes accurately reflected their experiences.

## 3 Results

### 3.1 Perceived positive experiences

The positive experiences perceived by the participants were categorized into three domains: emotional reactions from meeting their childhood self, shifting attitudes toward the self, and the advantages of using VR. Nine themes were identified across the domains. The results are summarized in Table 4.

**Table 4:** Perceived positive experiences.

Domain	Theme
Emotional reactions from meeting my childhood self	Feeling overwhelmed by resurfacing unresolved childhood memories
	Feeling compassion for my younger self
	Feeling proud of how far I have come
Shifting attitudes toward the self	Negative emotions toward myself replaced with positive ones
	No longer blaming myself
	Becoming more accepting of myself
Advantages of using VR	Being fully immersed in the “empty chair” conversation
	Not having to worry about the counselor’s gaze
	Being able to vividly recall my childhood self

#### 3.1.1 Emotional reactions from meeting my childhood self

This domain included four themes related to the participants’ immediate emotional responses during the VREC technique, particularly as they encountered and interacted with the virtual representation of their younger selves. The first theme, “feeling overwhelmed by resurfacing unresolved childhood memories,” captured the intensity of emotions that participants experienced as they recalled difficult situations from their childhood. Many described feeling more emotionally overwhelmed than they had anticipated, experiencing a heavy heart, or almost being brought to tears. For example, Participant 1 shared that simply facing the child in VR conveyed “so much pain… even without words,” making it difficult to begin speaking. Similarly, Participant 8 noted, “It felt like I was seeing my younger self all over again,” describing how vivid memories of being scolded and discouraged suddenly returned, leaving them emotionally choked (Table 5).

**Table 5:** Perceived negative experiences.

Domain	Themes
Difficulty revisiting childhood without constraints	Challenges in recalling various aspects of my childhood self
	Difficulty connecting with emotions from childhood
Elements that disrupted immersion	Mismatch between virtual representation and my actual self
	Unfamiliarity with VR and HMD
	Limited nonverbal expression of the virtual human
	Difficulty with self-disclosure
Areas for improvement	Increasing the variety of virtual humans and backgrounds
	Improving the level of interaction with the virtual human
	Allowing more counseling time to build rapport

The second theme, “feeling compassion for my younger self,” reflected participants’ experiences of feeling sorry for and kindness toward their past selves. These emotions arose when they reflected on how difficult their childhood had been and how helpless they had felt during that period. Participant 3 expressed regret, stating, “It was pitiful… heartbreaking. I wished someone had been there to care for me.” Participant 5 similarly noted, “I was shy and withdrawn… I lacked confidence. After my father hit me, I became more passive,” adding, “I just felt sorry for myself. If the character in the VR had been real, I would have hugged him silently without saying a word.”

The third theme, “feeling proud of how far I have come,” captured participants’ sense of accomplishment and pride in overcoming the challenges of their difficult childhood and growing into the people they are today. For example, one participant shared, “Even though I was like this as a child, I feel like I have done well to make it this far. Despite the hardships and long periods of pain, I managed to keep going. I felt a sense of pride in myself. I felt like I wanted to support myself” (Participant 9).

#### 3.1.2 Shifting attitudes toward the self

This domain included three themes associated with changes in participants’ attitudes toward themselves that occurred after engaging in dialog with their younger selves. The first theme is “negative emotions toward myself replaced with positive ones.” Participants initially reported feeling overwhelmed and sorry while recalling their childhood. However, when they expressed these emotions as virtual representations of their younger selves, they gradually experienced relief and pride. For example, Participant 8 shared, “During this conversation, I realized the words I had been holding inside… After doing this, my heart feels slightly lighter. Although regretful, it also feels good to let this out. It feels like a weight has been lifted off my shoulders.”

The second theme, “no longer blaming myself,” described a turning point in participants’ self-perception, as they came to realize that the hardships they experienced during childhood were not their fault. This understanding emerged as they spoke words of reassurance to their younger selves, as Participant 10 shared, “I always thought it was my fault… But when I told myself, ‘You did not do anything wrong,’ instead of hearing it from someone else, it brought up emotions I had never felt before.”

The third theme, “becoming more accepting of myself,” reflected participants’ experiences of recognizing and accepting their present selves more fully through the empty-chair technique. An example of this theme is as follows:

“It is me, in the present, comforting myself. After saying a few words, I feel like I have begun to let go of the inferiority that I have held. I decided to live as I am now without constantly comparing myself with others. What I said to my younger self is what I am saying to myself now.” (Participant 9).

#### 3.1.3 Advantages of using VR

The first theme, “being fully immersed in the empty-chair conversation,” describes how the presence of a visible virtual figure enhanced participants’ focus and emotional engagement. Participant 12, who had previously used the empty-chair technique as a counselor, shared, “I have often asked clients to imagine someone sitting in the chair, but after doing it myself today, I realized that imagination alone makes it hard to vividly recall emotions. Seeing the figure helped me truly reflect on my childhood and speak more comfortably.” Similarly, Participant 14 said, “Having a figure in front of me made it easier to talk. If I were just facing an empty chair, it would have felt like I was talking to a ghost. But because I did not have to force myself to imagine someone, I was able to open up and say things I normally would not.”

The second theme, “not having to worry about the counselor’s gaze,” highlights the sense of psychological safety that participants felt when visually separated from the counselor by the HMD. This setup allowed them to feel alone with their younger selves, thereby reducing their self-consciousness and encouraging openness. As Participant 4 noted, “It felt like I was just with the virtual character. When you meet a counselor for the first time or do not know them well, you can feel self-conscious. But in this setup, the feeling of being watched fades, which really helped me open up.” Similarly, Participant 9 shared, “If I had used a regular empty chair, I would have still felt someone was watching nearby. But here, it truly felt like just me and my childhood self. That sense of privacy helped me focus on what I wanted to say.”

The third theme, “being able to vividly recall my childhood self,” illustrates how the visual details of the virtual boy, such as facial expressions, posture, clothing, and hairstyle, evoked strong memories of participants’ early experiences. Participant 1 shared, “At first, I noticed things like the clothes or face, but then I saw the slouched shoulders, dirt on the neck, even the way the feet were positioned. I kept thinking, ‘That was me.’ I remembered avoiding eye contact, fidgeting, and swaying—those behaviors were mine.” Participant 2 similarly noted, “The stretched neckline of the t-shirt stood out. As a child, I wore that kind of shirt all the time, because I had no choice. I remembered trying to cover it up when my classmates noticed and made excuses such as, ‘It is just the design.’ That small detail brought all those memories back.”

#### 3.1.4 Difficulty revisiting childhood without constraints

This domain included two themes that reflected the challenges faced by participants in recalling their childhood experiences and emotions owing to the presence of virtual representations. The first theme, “challenges in recalling various aspects of my childhood self,” captures participants’ cognitive difficulties in retrieving specific autobiographical details, such as their past appearance, behaviors, or contextual memories. Although the VR environment was designed to support memory recall through visual cues, several participants noted that the preconstructed virtual representation, particularly when it did not align closely with their personal history, hindered their ability to access certain memories. For example, Participant 5 noted, “The visual presence of a virtual character is helpful in some ways, but it also feels somewhat restrictive. Compared to closing my eyes and imagining my childhood self, I found it harder to access specific memories.”

In contrast, the second theme, “difficulty connecting with emotions from childhood” refers to cases in which participants were able to recall past events but struggled to reconnect with the emotions tied to those experiences. Some participants found that the visual specificity of the virtual representation, rather than deepening emotional engagement, limited their ability to reconnect with the intensity of their past feelings. As Participant 5 elaborated, “I found it harder to access the vivid emotions tied to them, such as the fear and sadness I felt during moments of violence. I think that the traditional method, which relies solely on imagination, may foster a deeper emotional connection.”

#### 3.1.5 Elements that disrupted immersion

The second domain, “elements that disrupted immersion,” addressed the factors that hindered participants’ immersion during the VREC technique. This domain included four themes. The first theme, “mismatch between virtual representation and my actual self,” referred to instances in which the appearance of the virtual representation differed from the participants’ actual childhood memories, making it difficult for them to fully immerse themselves in the process. An example of this theme is as follows:

“I remember being naked or in my underwear when I am chased out of the house. If the virtual boy had been dressed like that, those memories would have been returned more vividly. But the t-shirt, jeans, and especially the canvas shoes made him look too well dressed, which made it harder to connect.” (Participant 2).

The second theme, “unfamiliarity with VR and HMD,” captured the awkwardness participants felt when using VR for the first time and the challenges they faced in operating the HMD and controllers, which did not work as smoothly as they had expected. For example, Participant 2 noted, “It was my first time using VR, and I found myself more focused on the novelty and how to control the device than on the emotional experience.” Similarly, Participant 3 shared, “Since I had never used VR before, it felt unfamiliar and awkward, and it did not quite feel real to me.”

The third theme, “limited nonverbal expression of the virtual representation,” referred to the limited range of nonverbal behavior of the virtual representation, which was restricted to actions such as avoiding eye contact or lowering his head. This limitation hindered participants’ immersion. As an example, Participant 4 stated, “The character kept repeating the same actions, such as lowering his head and then looking up, over and over again. These repetitive and simple movements seemed to disturb my immersion, especially at the beginning.”

The fourth theme, “difficulty with self-disclosure,” pertained to participants’ personal characteristics, specifically their discomfort with sharing personal stories in front of others. In this case, participants had access to both memories and associated emotions, but experienced discomfort in verbally expressing these experiences, especially in the presence of others. Participant 5 shared that some memories had never been disclosed, not even to close family members, and expressed how difficult it was to speak about something known only to himself: “There are parts of my past I have never shared—not even with my wife. It is not easy to bring that out.” Similarly, Participant 14, reflecting on his personal and professional identity, said, “As a lawyer and man, I have never expressed my emotions. Doing so now felt strange, like going against a habit I have had all my life.”

#### 3.1.6 Areas for improvement

The third domain, “areas for improvement,” addressed aspects of the VR-based empty-chair technique that the participants felt required improvement. This domain included three themes. The first theme, “increasing the variety of virtual representation and backgrounds,” highlighted the need for greater diversity in virtual representation and backgrounds to enhance immersion and realism. Participant 6 suggested that using childhood photos to generate virtual representation could create a more emotionally resonant experience: “If a virtual human could be created based on childhood photos, it might feel more personal and effective.” Participant 13 also expressed a desire for more customization options, such as recreating their childhood room or having a wider range of virtual appearances: “The customization was nice, but I wish it were more diverse.”

The second theme, “improving the level of interaction with the virtual representation,” reflected the need for the virtual representation to display more natural nonverbal behavior and respond verbally, as these improvements could enhance immersion. Participant 3 suggested that simple verbal responses from the virtual figure could ease the interaction and help them focus: “Having it respond to questions, even just by voice, might make it easier to interact.” Participant 10 noted the lack of eye contact as a barrier to emotional connection, saying, “Even brief moments of eye contact could have helped me connect more emotionally.”

Finally, “allowing more counseling time to build rapport” referred to the suggestion that dedicating sufficient time to establish rapport with the client before implementing the VR-based empty-chair technique would be beneficial. Participant 4 reflected, “I hesitated and ended up speaking indirectly because the time felt too short. The length or frequency of sessions definitely affects how much I can open up.” Similarly, participant 11 noted, “Spending more time building connection with the counselor could have helped me feel more understood and engage more deeply in the process.”

### 3.2 IPQ

As shown in Table 6, the IPQ results indicate that the mean scores for presence, immersion, and realism are approximately 4 on a 7-point scale.

**Table 6:** The results of IPA.

Subscales	*M*	*SD*
Presence	4.465	1.620
Immersion	4.821	1.559
Realism	4.410	1.783

## 4 Discussion

Considering previous findings that clients may perceive traditional imagery-based empty-chair techniques as awkward or uncomfortable (Lee, 2013) and that middle-aged South Korean men often struggle with self-disclosure and emotional expression in counseling (Lee and Kim, 2024), this study explores the positive and negative experiences of middle-aged South Korean men using a VR-based empty-chair technique. We discuss the major findings in relation to our research questions and existing literature.

### 4.1 Major findings

Participants reported that interacting with a virtual representation helped them recall their childhood selves and elicited strong emotional responses. First, visual cues such as the virtual representation’s shabby attire and nonverbal behaviors (e.g., lowered head and averted gaze) played a key role in reconnecting participants with childhood memories. These cues evoke self-compassion and empathy, making the experience particularly impactful. Additionally, the tangible presence of the virtual representation facilitated self-disclosure and emotional expression, which the participants found easier than the traditional empty-chair technique, which relies solely on imagination.

This finding is particularly significant because the participants were middle-aged South Korean men who often struggled with self-disclosure and emotional expression (Lee and Kim, 2024). Through this process, participants reported experiencing a sense of relief and catharsis that they had rarely encountered. Interestingly, although the virtual representation’s face did not resemble the participant’s childhood appearance, other visual and contextual cues effectively aided in the recall and expression of childhood emotions.

However, some participants noted the limitations of the VR environment. Although visual cues are generally helpful, they restrict the ability to recall specific childhood memories. This suggests that the effectiveness of these cues may depend on individual differences, such as participants’ capacity for imagination. Specifically, the limited range of nonverbal behaviors of virtual representations made it harder for some participants to evoke childhood experiences and connect deeply with the associated emotions. These findings align with those of previous studies on VR-based interventions such as VRET and VR-based mindfulness. For example, VR technology is effective for individuals who struggle to vividly imagine anxiety-inducing situations (as in VRET) or calm natural environments (as in VR-based mindfulness) (Jo et al., 2024). Therefore, having a discussion with clients beforehand is crucial to determine whether a traditional imagery-based empty-chair technique or VR-based approach aligns better with their needs and preferences.

Second, the visual cues provided by VR and the use of an HMD significantly enhanced the immersive experience. This is consistent with previous studies suggesting that VR reduces awareness of real-world distractions, such as the presence of researchers or counselors, by using an HMD that dynamically adjusts the virtual environment based on head movements through head tracking (Kim and Kim, 2022; Nam et al., 2017). Participants noted that the immersive VR experience reduced their psychological burden and facilitated their self-disclosure. This observation aligns with that of previous studies showing that clients experience less tension and pressure when they are not physically present with a counselor, which promotes more comfortable self-expression (Lee et al., 2015).

Third, participants reported reduced self-blame and increased self-acceptance when using the VREC technique. Similar findings have been observed in studies on the traditional empty-chair technique, in which individuals often develop a deeper understanding and acceptance of their childhood experiences, thereby reducing their self-blame (Lee, 2013; Pugh and Salter, 2021). These findings also reflect the paradoxical theory of change, which highlights the irony that true change occurs only when individuals fully accept painful emotions as they are (Greenberg and Malcolm, 2002; Paivio and Greenberg, 1995). In this study, the VR-based setting facilitated emotional acceptance by enabling participants to visually confront their childhood selves in a private environment. The immersive experience, combined with the reduced awareness of external judgment provided by the HMD, allowed participants to remain with and express difficult emotions more openly. This emotional engagement reflects a paradoxical process, in which the acceptance of distressing feelings becomes a catalyst for self-understanding and change. Although the brief VR-based intervention may not have fully resolved these emotions, participants reported increased self-compassion and emotional relief, highlighting the potential of VR to foster meaningful shifts even in short-term work.

Finally, although the participants reported positive experiences with the VREC technique, they also identified several negative aspects primarily associated with technical limitations. Specifically, they encountered difficulties using the HMD device and controllers and reported issues with the virtual representation’s limited nonverbal behaviors, which hindered immersion. To address these challenges, the participants suggested enhancing the virtual representation’s ability to exhibit more natural nonverbal cues and respond verbally. They also recommended allocating additional time during counseling sessions to establish a stronger rapport with the counselor when implementing the VR-based technique in practice. These findings resonate with previous studies highlighting the critical role of counselor-client rapport and counselors’ active empathy in resolving unresolved issues using the empty-chair technique (Berdondini et al., 2012; Kim and No, 2002).

To address the third research question, the results from the IPQ indicated moderate levels of presence, immersion, and realism in the VREC technique, with average scores in the 4-point range on a 7-point scale. Immersion scored the highest, suggesting that participants generally felt engaged in the virtual environment. This finding can be understood considering the definition and measurement of immersion in the IPQ, which refers to the degree of mental absorption and attentional focus on a virtual experience, regardless of whether the environment is perceived as fully realistic or spatially convincing (Schubert, 2003). The findings on IPQ, particularly the relatively high immersion scores, align with qualitative themes such as “being fully immersed in the empty-chair conversation” and “not having to worry about the counselor’s gaze,” which reflect participants’ reports of feeling absorbed in the VR setting and being more open to emotional expression due to reduced self-consciousness enabled by HMD. By contrast, presence and realism received relatively lower scores, which aligns with participants’ accounts of “mismatch between virtual representation and my actual self” and “limited nonverbal expression of the virtual representation.” These themes describe how certain visual and behavioral aspects of the VR character did not fully match their expectations or memories, thereby hindering their emotional connections. Additionally, “unfamiliarity with VR and HMD” helps explain how technological novelty may have interfered with some participants’ ability to fully immerse themselves, particularly those with no prior VR experience.

The IPQ scores suggest that, although the VR-based technique successfully created an engaging environment for participants, there is room for improvement in enhancing the sense of presence and realism. Factors such as the virtual representation’s limited nonverbal expressions or participants’ unfamiliarity with VR technology may have contributed to these moderate scores. Improvements in visual and interactive elements, including more realistic virtual representations and customizable settings, could enhance these aspects and foster greater emotional engagement during sessions. More importantly, these technical limitations may have compromised the emotional depth of the intervention. For example, when nonverbal cues were perceived as repetitive or unnatural, participants reported difficulty sustaining emotional connections, which could diminish the therapeutic potential of the exercise. Similarly, for first-time users, the novelty and discomfort of using an HMD distracted them from the internal focus required for self-reflection and emotional processing. These issues suggest that unless such limitations are addressed, VR-based techniques may not achieve their full potential as a complement to traditional methods.

Taken together, these findings contribute to the broader literature by demonstrating how VR can extend the principles of Gestalt therapy—such as emotional awareness and self-dialog—into immersive formats. By enabling participants to externalize and re-engage with difficult emotions in a visually embodied way, VR-based interventions may provide a novel modality for clients who struggle with traditional, imagination-based methods. These results should be interpreted cautiously, as they reflect the experiences of a small group of middle-aged South Korean men, and may not be generalizable to other populations. Nonetheless, the study suggests that VR technology has the potential to complement and possibly enhance key mechanisms of Gestalt interventions, particularly among populations where emotional expression is culturally constrained.

This study offers several contributions. First, it applies a VR-based Gestalt intervention to middle-aged South Korean men, a group at high risk for mental health difficulties but rarely represented in VR therapy research. Second, it uses a standardized younger-self avatar that reflects distress rooted in patriarchal father–child relationships, providing a culturally relevant adaptation of the empty-chair technique. Third, unlike most VR studies that emphasize pre–post symptom reduction, this study focuses on participants’ narratives from in-depth interviews. These qualitative experiences provide insight into the subjective and emotional processes of VR-based experiential work that are often overlooked in quantitative research.

### 4.2 Implications for clinical practice and technology

This study has several tentative implications for clinical practice. Visual cues provided through VR may help facilitate the process of recalling childhood memories and expressing related emotions. These results, along with those of previous studies that have reported promising findings regarding VR-based empty-chair techniques (Brahnam, 2014; Ganschow et al., 2021), suggest that VR could serve as a potentially valuable tool in therapeutic settings. To maximize its benefits, clinicians may consider familiarizing themselves with the VR implementation, including structuring the process, using relevant devices, and addressing clients’ potential discomfort. However, as visual cues in VR have also been reported to sometimes hinder memory recall (Kim and Kim, 2023), clinicians should discuss the possible advantages and disadvantages of both traditional and VR-based techniques with patients beforehand, allowing them to make informed decisions.

Furthermore, clients who are unfamiliar with the virtual environment or inexperienced with HMDs and controllers might find it challenging to fully immerse themselves (Matamala-Gomez et al., 2019). Providing adequate preparation time may help clients become more comfortable with the equipment, and a virtual representation can be essential. Counselors can further reduce anxiety and foster a sense of psychological safety by discussing specific topics to be explored during the sessions and setting clear expectations (Maples-Keller et al., 2017). They should remain attentive to signs of emotional distress such as withdrawal or heightened agitation and provide tailored support as needed. This could include offering reassurance, encouraging breaks, or shifting the focus to less emotionally intense topics. If required, counselors can also adjust the session by transitioning to less immersive techniques, such as verbal discussions or imagery-based exercises, to ensure that the client feels supported and engaged (Maples-Keller et al., 2017). Moreover, to prevent cybersickness from prolonged use of the HMD, VR-based empty-chair sessions may be best limited to 15–30 min, depending on individual tolerance.

### 4.3 Implications for technical improvements

This study highlights the need for technological advancements in VR-based empty-chair techniques. A key development area involves the creation of virtual representations capable of responding both verbally and nonverbally. By integrating AI technologies and leveraging accumulated data, these virtual entities offer more dynamic and interactive experiences, which may foster deeper emotional engagement and potentially contribute to improved therapeutic processes.

Another possible improvement involves diversifying the background settings of the virtual environment to better reflect the clients’ childhood contexts. For example, incorporating scenes such as school classrooms, playgrounds, family living rooms, or parks—common settings in the childhood of middle-aged men—might help make the experience more relatable and emotionally impactful. Tailoring these environments to align with clients’ specific memories could enhance their sense of realism and connection, thereby supporting the therapeutic process.

### 4.4 Limitations and directions for future research

This study has several limitations and suggestions for future research. First, we used a single standardized virtual representation instead of creating personalized virtual characters that reflect each participant’s childhood appearance. This approach was chosen to ensure experimental consistency and control for variables that could arise from different visual representations. By standardizing the virtual representation, this study aimed to focus on the broader effects of the VREC technique rather than on individual differences in character design. However, the degree of resemblance to participants’ childhood selves may influence their experiences, which future studies can investigate. Therefore, future research should explore how the use of personalized virtual characters may yield results that differ from those obtained in this study. Second, this study focused exclusively on middle-aged South Korean men to understand the impact of cultural norms and gender roles on VREC techniques in this specific group. Although this focus provides valuable insights, it does not capture the full range of individual differences and socioeconomic variability within this demographic. Additionally, the exclusion of other groups such as women and younger adults limits the generalizability of our findings. Future research should include a more diverse participant pool to explore the effectiveness and acceptance of the technique across various demographics. Moreover, given that emotional expression and self-disclosure are shaped by cultural expectations, the mechanisms through which VR facilitates emotional engagement may vary across cultural contexts. Therefore, cultural adaptation may be required when applying this technique to populations with different emotional norms or therapeutic expectations.

Third, this study was limited by its small sample size and implementation of the VREC technique in only one session. Furthermore, most participants reported psychological distress rooted in emotionally distant or patriarchal father figures, which, although culturally relevant in the South Korean context, further narrowed the scope of the findings. These factors limit the generalizability of the results to all middle-aged men in diverse real-world counseling contexts. Future studies should recruit larger and more diverse samples, including different gender identities, age groups, and family dynamics, and employ multiple sessions to comprehensively examine the process and therapeutic outcomes of the VREC technique. Fourth, this study did not compare the VREC technique with the traditional imagery-based empty-chair techniques. Future studies should address these limitations by conducting comparative analyses to determine the relative effectiveness and advantages of each approach. A mixed-methods design may be particularly useful in this context, as quantitative measures (e.g., symptom reduction, increases in self-compassion, or reductions in self-blame) could assess treatment outcomes, whereas qualitative interviews could explore participants’ subjective experiences, perceived emotional depth, and engagement. This approach would allow for a more comprehensive understanding of how each method supports emotional expression and therapeutic changes. Fifth, while the use of IPQ was validated for general VR contexts, it is not specifically designed for therapeutic applications. Thus, it may not fully capture clinically relevant aspects of presence, such as emotional resonance or perceived interpersonal connection with virtual representations. Future research should consider supplementing or comparing IPQ with other presence or engagement measures tailored to clinical VR experiences. Finally, although participants generally responded positively to the VREC technique, the potential emotional risks associated with revisiting unresolved childhood experiences in immersive settings must be acknowledged. Some participants reported temporary emotional distress during or immediately after the sessions. All sessions were conducted by graduate students in counseling psychology under the supervision of a licensed clinical psychologist. After each session, participants received emotional debriefing and grounding and were provided with information for follow-up counseling support if needed. Future research should continue to monitor and report on emotional safety protocols, particularly when using VR to facilitate the recall of painful or emotionally sensitive memories.

## 5 Conclusion

Despite these limitations, this study provides preliminary insights into the potential use of VR-based empty-chair techniques among middle-aged South Korean men, a group that often faces cultural and personal barriers to emotional expression. By enabling participants to reconnect with their childhood selves, the technique appeared to facilitate the experience and expression of emotions, such as sadness and regret, which in turn were associated with reduced self-criticism, increased self-acceptance, and positive emotional shifts. These findings should be interpreted cautiously and limited to the specific cultural and demographic group studied. They suggest that VR-based empty-chair techniques may serve as a potentially useful alternative for middle-aged South Korean men who struggle with traditional approaches, particularly when counselors establish a strong rapport with clients beforehand. As related technologies continue to evolve, the effectiveness and accessibility of such interventions may improve, but further research is needed to test their applicability in other populations and contexts. In addition, this study provides initial insights into the early development and feasibility of VR content in psychotherapy, highlighting its potential applications while acknowledging the need for additional empirical validation.

## Data Availability

The raw data supporting the conclusions of this article will be made available by the authors, without undue reservation.
